# Spontaneous Rupture of Uterus in Midtrimester Pregnancy Due to Increased Uterine Pressure with Previous Laparoscopic Myomectomy

**Published:** 2013-09-18

**Authors:** Mine Kiseli, Hakan Artas, Figen Armagan, Zeynep Dogan

**Affiliations:** 1 Department of Obstetrics and Gynecology, Faculty of Medicine, University of Ufuk, Ankara, Turkey; 2 Department of Obstetrics and Gynecology, Elazıg Training and Research Hospital, Elazıg, Turkey; 3 Department of Radiology, Elazıg Training and Research Hospital, Elazıg, Turkey

**Keywords:** Uterine Rupture, Uterien Myomectomies, laparoscopy, Midtrimester

## Abstract

Uterine rupture is a life threatening condition for both the mother and her fetus. It may be seen in the second trimester usually after induction for pregnancy termination in a scarred uterus. Spontaneous rupture in the second trimester before labor is a very rare condition. Here, we report a case of uterine rupture at 23-week pregnancy due to elevated uterine pressure with a history of laparoscopic myomectomy one year before admission. She was symptomatic for diffuse abdominal pain and the ultrasonographic image was interpreted as amniotic band. Four days later, because of deterioration of the patient and fetal bradycardia urgent laparotomy was performed. Fundal rupture with fibrotic borders suggested that a chronic event was seen. Laparoscopic myomectomy has advantages over laparatomy but the possibility of uterine rupture in following pregnancies should not be underestimated. Therefore, repair of the myometrium should be carefully assessed.

## Introduction

Uterine rupture, defined as disruption or tear of the myometrium and serosa of uterus, is a life threatening condition for both the mother and the fetus. Previous caesarean scar or myomectomy, trauma, grand-multiparity, uterine anomaly or injudicious use of oxytocin or prostaglandin are some of the predisposing factors responsible for rupture of the uterus during labor. Rupture of the uterus may also be seen in the second trimester, occurring after induction for pregnancy termination in scarred uterus most of the time. The incidence of uterine rupture at second trimester pregnancy termination by misoprostol is reported at 0.4% with one prior low transverse cesarean delivery (1).

Spontaneous rupture of the uterus in the second trimester is very rare. Placenta percreta as well as scar pregnancy have been thought as predisposing factors of spontaneous midtrimester uterine rupture. But without any medication for induction and placenta percreta, spontaneous rupture in midtrimester is a noteworthy condition. Here we report a prolonged uterine rupture in midtrimester *in vitro* fertilization (IVF) pregnancy, with a history of laparoscopic myomectomy, misdiagnosed initially as amniotic band at ultrasonography. 

## Case Report

**Fig 1:**
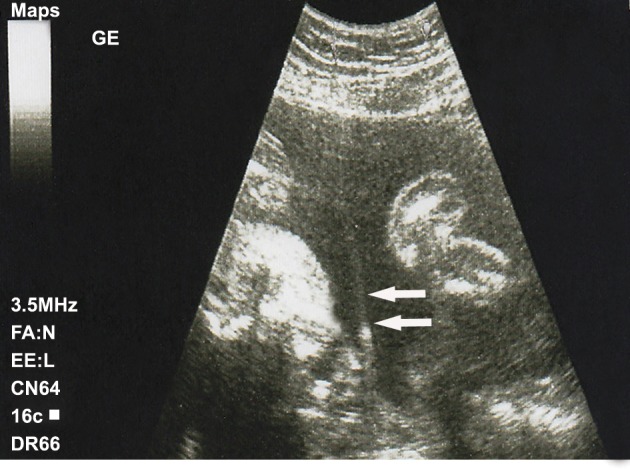
Ultrasonographic image of the patient at second admission, interpreted as amniotic band.

A 33-year-old pregnant woman was admitted to the emergency department of our clinic with acute diffuse abdominal pain. Her history revealed infertility for 4-5 years and laparoscopic myomectomy performed for a single 3 cm subserous myoma located in the fundus, one year ago in another institution. At laparoscopic myomectomy, hemostasis had been supplied by electrocoagulation and no suturing was performed. Six months after the operation, she successfully underwent IVF. On admission she was at 23 weeks gestation according to her last menstrual period and the ultrasonography revealed 23 weeks gestation with fetal heart beat and posterior located placenta. No contractions were detected on the tocogram. Her blood tests including hemoglobin, white blood cell count, liver enzymes and urine analysis were normal. The cervix was closed and 35 mm in length based on ultrasonography. Because of a possible diagnosis of preterm labor, intravenous hydration was performed and the abdominal pain regressed gradually. The patient was discharged 4 days later. Few hours after discharge, she was readmitted with abdominal pain. In this instance, an unusual image as amniotic band on fundus uteri was detected by ultrasonography (Fig 1). Her vital signs were normal (pulse: 82/minute and blood pressure: 120/70 mmHg) and no pelvic tenderness was found. She was rehospitalized and intravenous hydration was applied. On the fourth day of her second hospitalization, her pain had progressed but neither cervical dilatation nor contraction was demonstrated. The same amniotic band image was seen on ultrasound. Acute appendicitis was excluded by general surgeons after evaluating the patient. A few hours later, after being examined by ultrasonography, severe fetal bradycardia was diagnosed. Moreover, she had guarding on physical examination which was not present before. Due to hypotension (BP: 90/50 mm Hg) and non-reassuring fetal heart rate, urgent laparotomy was decided on the 24^th^ week of gestation. During operation, fundal rupture of the uterus with the amniotic sac in the abdomen was seen. About 500 ml of free blood was present in the abdominal cavity. A uterine border of the rupture area was fibrotic suggesting chronic rupture. After evacuating the fetus and placenta, fundal rupture was repaired with three layers of sutures and one package of red blood cells was transfused. Postoperative follow-up was unremarkable and the patient was discharged on the third postoperative day.

## Discussion

Myomectomy can be performed in unexplained infertility cases with only myoma uteri in the female partner. The localization of the fibroids is important for fertility. Submucous myomas are the most problematic ones causing implantation problems and repeated miscarriages. However, there are conflicts about relationship between intramural, subserous fibroids and infertility. Pregnancy rates after myomectomy were reported approximately 50% in infertile patients (2). Laparoscopic repair has been widely used in recent years due to improvements in the techniques. The advantages of laparoscopic myomectomy in infertile patients include short recovery time, less pain and less adhesion formation. Nevertheless, there is still a debate about the strength of the uterine repair. First of all, location and size of myomas are important in deciding the surgical approach as well as surgical skills of the surgeon. Patients with more than four or larger than 8-10 cm myomas are not good candidates for laparoscopic surgery (3). Preoperative ultrasound and MRI are useful diagnostic tools for evaluating the localization, number and size of the myomas; aiding the surgeon in deciding on the operation technique.

Preoperative medical treatment with gonadotropin-releasing hormone (GnRH) agonists have been known to reduce the size of the myoma, thus reducing the blood loss in the operation (4). However, long term postoperative complications such as recurrence and uterine rupture have not been clearly understood (5).

One of the important complications after laparoscopic myomectomy is uterine rupture during pregnancy. The incidence of uterine rupture in following pregnancies after laparoscopic myomectomy is reported at 1% by Dubuisson et al. (6). Although the risk is not so high with an optimum technique, myomectomy involving the endometrium is a risk factor for uterine rupture. Scar healing is an important issue for uterine rupture which can be evaluated after laparoscopy. There are studies showing the effectiveness of ultrasonography for assessing uterine scars after myomectomy. Traditional two dimensional ultrasound examination of uterine scar and also Doppler velocimetry and resistance index of the uterine arteries were used to follow up scar healing and strength (7). Dubuisson et al. (6) stated that second look laparoscopy may be useful in assessing the thickness and quality of the hysterotomy scar. Moreover, early pregnancy ultrasound assessment after laparoscopic myomectomy should be performed especially with attention to localisation of placenta to exclude scar pregnancies.

As far as the authors know, very few cases of spontaneous uterine rupture after laparoscopic myomectomy in second trimester have been reported after 2002 (8, 9, 10). This may be due to the improvement in surgical skills and techniques recently, but we should always be careful while deciding laparoscopic technique. Good approximation with appropriate sutures without hematoma formation is essential even in superficial myomectomies (6). Intraoperative transvaginal ultrasound is an option to examine the haematomas deep in the myometrium since large intramural myomas are not good candidates for laparoscopy. Laparotomy should be preferred if the repair would not be done well; and laparoscopies should be performed by high skilled laparoscopic surgeons in specialized centers.

Use of electrocoagulation during myomectomy in our case emphasizes the long-term consequences of electrical energy. Hasbargen et al. (11) reported a case of uterine rupture in a small pedunculated and subserosal myoma resected by electrocoagulation only. They told that even in the absence of uterine incision, electrocoagulation can lead to uterine weakness and uterine rupture in following pregnancy (11). Pelosi et al. (12) also reported rupture in a woman with a history of superficial subserous laparoscopic myomectomy. In young patients uterine suturing is highly recommended especially in cases with future fertility desire.

This is the first case of early second trimester spontaneous uterine rupture who was clinically silent for four days until placental abruption occurred. As the patient presented with nonspecific symptoms, the differential diagnosis was harder. The only risk factor was previous laparoscopic myomectomy, but the time of rupture is noteworthy. The ultrasound evaluation during the second hospitalization of the patient lead to misdiagnosis thought to be amniotic band. In pregnant patients presenting with nonspecific abdominal pain, the obstetrician should keep in mind the probability of silent rupture if there is a history of laparoscopic myomectomy. MRI may be helpful in accurate diagnosis for such cases as reported by Hasbargen et al. (11).

Preoperative evaluation of infertile patients with myomas should be extensive (evaluation). Electrocoagulation may be dangerous even in superficially located small myomas; methods other than surgery for myomectomy such as MR-guided focused ultrasound are new alternatives for these patients. Infertile women should be carefully assessed and a discusssion about the advantages and possible complications of the treatment should be made.

## References

[1] Berghella V, Airoldi J, O’Neill AM, Einhorn K, Hoffman M (2009). Misoprostol for second trimester pregnancy termination in women with prior caesarean: a systematic review. BJOG.

[2] Desai P, Patel P (2011). Fibroids, infertility and laparoscopic myomectomy. J Gynecol Endosc Surg.

[3] Holub Z (2007). Laparoscopic myomectomy: indications and limits. Ceska Gynekol.

[4] Chen I, Motan T, Kiddoo D (2011). Gonadotropin-releasing hormone agonist in laparoscopic myomectomy: systematic review and meta-analysis of randomized controlled trials. J Minim Invasive Gynecol.

[5] Fedele L, Vercellini P, Bianchi S, Brioschi D, Dorta M (1990). Treatment with GnRH agonists before myomectomy and the risk of short-term myoma recurrence. Br J Obstet Gynaecol.

[6] Dubuisson JB, Fauconnier A, Deffarges JV, Norgaard C, Kreiker G, Chapron C (2000). Pregnancy outcome and deliveries following laparoscopic myomectomy. Hum Reprod.

[7] Tinelli A, Hurst BS, Mettler L, Tsin DA, Pellegrino M, Nicolardi G (2012). Ultrasound evaluation of uterine healing after laparoscopic intracapsular myomectomy: an observational study. Hum Reprod.

[8] Grande N, Catalano GF, Ferrari S, Marana R (2005). Spontaneous uterine rupture at 27 weeks of pregnancy after laparoscopic myomectomy. J Minim Invasive Gynecol.

[9] Goynumer G, Teksen A, Durukan B, Wetherilt L (2009). Spontaneous uterine rupture during a second trimester pregnancy with a history of laparoscopic myomectomy. J Obstet Gynaecol Res.

[10] Torbé A, Mikołajek-Bedner W, Kałużyński W, GutowskaCzajka D, Kwiatkowski S, Błogowski W (2012). Uterine rupture in the second trimester of pregnancy as an iatrogenic complication of laparoscopic myomectomy. Medicina
(Kaunas).

[11] Hasbargen U, Summerer-Moustaki M, Hillemanns P, Scheidler J, Kimmig R, Hepp H (2002). Uterine dehiscence in a nullipara, diagnosed by MRI, following use of unipolar electrocautery during laparoscopic myomectomy. Hum Reprod.

[12] Pelosi MA 3rd, Pelosi MA (1997). Spontaneous uterine rupture at thirty-three weeks subsequent to previous superficial laparoscopic myomectomy. Am J Obstet Gynecol.

